# Clinical Efficacy in Relieving Dentin Hypersensitivity of Nanohydroxyapatite-containing Cream: A Randomized Controlled Trial

**DOI:** 10.2174/1874210601812010572

**Published:** 2018-08-31

**Authors:** Bennett T. Amaechi, Kelly C. Lemke, Shyamali Saha, Jonathan Gelfond

**Affiliations:** 1 Department of Comprehensive Dentistry, University of Texas Health Science Center, San Antonio, Texas, USA; 2 Department of Developmental Dentistry, University of Texas Health Science Center, San Antonio, Texas, USA; 3 Department of Epidemiology and Biostatistics, University of Texas Health Science Center, San Antonio, Texas, USA

**Keywords:** Dentifrice, Dentin hypersensitivity, Dentin tubules, Nanohydroxyapatite, Silica, Tubule occlusion

## Abstract

**Objective:**

The study aimed to investigate the effectiveness of Apadent Pro (Sangi) Nanohydroxyapatite (nHAP) dental cream to relieve Dentin Hypersensitivity (DHS), compared with a positive control cream containing 20% pure silica (Silica).

**Methods:**

In this double-blind, randomized, parallel-group clinical trial, patients diagnosed with DHS and qualified to participate were randomized into two groups, nHAP (n=25) and Silica (n=26). Subjects’ baseline and posttreatment sensitivity were assessed using two pain scales, a four-point Dental Pain Scale (DPS) followed by a linear Visual Analog Scale (VAS), after the application of ice-cold and air stimuli. Subjects used custom-fabricated trays to apply their respective cream for 5 minutes once daily following brushing with standard fluoride toothpaste. Posttreatment sensitivity (efficacy) was assessed every 2 weeks for 8 weeks. Mean treatment outcomes (percentage change from baseline) at each time point were compared using the Tukey HSD test for multiplicity (*P*<0.05).

**Results:**

With either air or cold stimulus, VAS and DPS indicated a significant (*P*<0.001) reduction in DHS at each time point with either nHAP or Silica. Comparing pain scales, VAS showed no significant difference in DHS reduction between the products with either air or cold. However, with DPS, DHS reduction was significantly (*P*<0.05) better with Silica than with nHAP at all time points with cold, and at 2, 4, and 8 weeks with air.

**Conclusion:**

Both Apadent Pro nHAP and Silica dental creams are effective at promoting the relief of DHS symptoms. When comparing the efficacy of the two compounds to relieve DHS, results of the two pain scales were conflicting.

## 1 INTRODUCTION

Dentin Hypersensitivity (DHS) is a relatively common clinical condition characterized by short, sharp pain that arises when exposed tooth dentin is subjected to external stimuli, typically thermal, evaporative, tactile, electrical, osmotic, or chemical, and which cannot be attributed to any other dental defect or disease. [1] These stimuli can be generated by the intake of certain food substances like ice-cream, hot tea/coffee, candies, fresh fruits, isotonic/energy drinks [2], oral hygiene procedures such as tooth-brushing [3], activities involving blowing air on teeth as can be experienced during dental examination, and [4] acids such as gastric acid in patients suffering from gastroesophageal reflux. Thus DHS is an exaggerated response that arises when a non-noxious stimulus affects dentin tubules exposed secondary to gingival recession, loss of enamel, or loss of the cementum layer that covers the root surface [2, 3]. The patient’s experience of DHS is physical and psychological discomfort that can range from minor annoyance to chronic irritation and has the potential to disrupt daily activities. In particular, DHS can affect the patient’s ability to eat, drink, and maintain good oral hygiene [4], and as such affects the individual’s quality of life.

The reported prevalence of DHS is higher in females than in males. In a United Kingdom study of 18 general dental practitioners, the ratio of female to male patients diagnosed with DHS was 1.5 to 1 [5]. DHS is reported in patients of all ages, but it is most often reported in the age group of 20 to 50 years, with peak prevalence from 30 to 40 years [6]. The most commonly affected teeth are the canines and premolars in both the maxilla and mandible. The cervical area of the buccal surface is the most affected tooth region [7].

The most widely held theory for DHS is the hydrodynamic theory, which postulates that when dentin tubules are open to the oral cavity due to enamel and/or cementum loss and a trigger stimulus occurs, fluid movement within the dentin tubules stimulates nerve receptors within the pulp, resulting in short, sharp pain [8].

The primary therapeutic approaches to DHS are desensitization through chemical agents that suppress or modify the nerve impulse, or occlusion of the dentin tubules to decrease dentin permeability and thereby prevent fluid movement in the dentin tubules [9]. Potassium ions, typically in the form of potassium nitrate, modify the nerve impulse by decreasing the excitability of the A fibers that surround the base of the odontoblastic process, resulting in a reduction in tooth sensitivity; however, evidence for the effectiveness of potassium nitrate-containing dentifrices in treating DHS is not clear [10].

Occlusion of the dentin tubules is the most common therapeutic approach [9]. Many of the agents in this category result in precipitative intratubular occlusion or in the formation of a smear layer over dentin that blocks entry to the tubules. None of these agents, however, have been established as a so-called gold standard for tubule occlusion. Professionally applied fluoride varnish treatment has been used extensively to reduce DHS, and while it has been shown in some studies to have a significant and immediate pain relief effect , [11, 12] two randomized controlled trials showed that the efficacy of 5% sodium fluoride varnish was not significantly different from that of the control varnish [13, 14]. Available evidence does not support the recommendation of products containing oxalate compounds or strontium chloride in the treatment of DHS, [15, 16] and studies on the efficacy of products containing Casein Phosphopeptide-Amorphous Calcium Phosphate (CPP-ACP) have been inconclusive [17-19]. More promising results have been reported for products containing silica-based bioglass, in particular calcium sodium phosphosilicate (NovaMin, Glaxo Smith Kline ) [20-23], and more recent studies have shown that pure silica has the ability to occlude dentin tubules [24-27].

Nanohydroxyapatite (nHAP) is another agent with the potential to treat DHS. Studies have shown that nHAP has the ability to remineralize caries when applied in toothpaste or topical cream form [28-38]. It is thought to maintain a state of supersaturation of calcium and phosphate at the tooth surface [39]. The potential of nHAP to promote crystal deposition and growth prompted a series of *in vitro* studies that established the potential of nHAP to occlude dentin tubules as a surrogate measure of its ability to relieve DHS symptoms [40-42]. These results prompted a further study using an in situ model, which also demonstrated that nHAP-containing toothpaste had the ability to physically occlude dentin tubules in human root specimens as a measure of their potential to treat DHS [43]. However, comparative clinical evidence is needed to establish the effectiveness of topically applied nHAP as a desensitizing agent. The aim of this clinical trial was to investigate the effectiveness of Apadent Pro (Sangi) nHAP dental cream to relieve DHS, comparing it with a positive control cream containing pure silica, in the form of combined standard pure silica particles and hydrated nanosilica.

It is pertinent to mention that DHS is a form of pain. Pain is a personal psychological experience, and its severity is known only to the sufferer, and an observer can play no legitimate part in its direct measurement [44]. For assessing response to treatment, a pain-relief scale has advantages over a pain scale. Pain cannot be said to have been relieved unless pain relief has been directly measured [44]. The Visual Analogue Scale (VAS), Numerical Rating Scale (NRS), Verbal Rating Scale (VRS), and the Faces Pain Scale-Revised (FPS-R) are among the most commonly used pain-relief scales for measuring pain intensity in clinical and research settings [45]. Although evidence supports their validity as measures of pain intensity, the VAS and NRS evidenced the most responsivity [45]. However, of the various methods for measuring pain, the visual analogue scale seems to be the most sensitive [44]. VAS is used to “quantify” the pain severity in millimeter (mm) in response to such query as “Draw a line on the scale that shows how much pain you have at this time”, with “no pain” on the far left end of scale at 0 mm and “pain as bad as it can be” on the far right at 100 mm. Similarly, NRS quantify pain severity in numbers 0-10. On the other hand, DPS is a scoring system that rates the pain intensity in response to the query “How much pain do you have at this time?”. Responses in this scale were assigned values from 0 (None) to 1 (mild), 2 (moderate), and 3 (Severe). Considering that DPS differs from both VAS and NRS, with VAS the most, the present study utilized both VAS and DPS for measurement of the DHS intensity overtime, after the application of two pain provocation tests, ice-cold and air stimuli.

This study tested six null hypotheses. The first null hypothesis was that VAS scores of the patients treated with Apadent Pro were not significantly different from those before treatment. The second null hypothesis was that DPS scores of the patients treated with Apadent Pro were not significantly different from those before treatment. The third null hypothesis was that VAS scores of the patients treated with pure silica were not significantly different from those before treatment. The fourth null hypothesis was that DPS scores of the patients treated with pure silica were not significantly different from those before treatment. The fifth null hypothesis was that there was no statistically significant difference between the VAS scores of patients treated with Apadent Pro and pure silica in relieving DHS. The sixth null hypothesis was that there was no statistically significant difference between the DPS scores of patients treated with Apadent Pro and pure silica in relieving DHS.

## 2 MATERIALS AND METHODS

This was a double-blind, randomized, parallel-group, single-center clinical trial to test the ability of dental cream containing 20% nHAP to relieve the symptoms of DHS, comparing it to that of a positive control cream containing 20% pure silica. The primary outcome to be examined was the percent change from baseline to air and cold stimulation at 2, 4, 6, and 8 weeks on a linear Visual Analog Scale (VAS) and a four-point Dental Pain Scale (DPS).

The study was conducted at the clinical research facility of the University of Texas Health Science Center at San Antonio (UTHSCSA) School of Dentistry. The UTHSCSA Institutional Review Board (IRB) approved the study (protocol #HSC20150221H), and the study was conducted in accordance with the ethical standards outlined in the 1964 Declaration of Helsinki and its later amendments. The study was registered with ClinicalTrials.gov (NCT02918617). Written informed consent was obtained from all participants prior to their participation in the study. The majority of participants were recruited from among patients receiving treatment in the dental school clinic and from among employees of UTHSCSA.

### 2.1 Participant Recruitment

Subjects aged 18 to 80 years were given a screening examination that included a medical history, oral examination, and DHS evaluation of teeth with exposed buccal cervical dentin. DHS was assessed by a positive response to a 2-second blast of air from an air-water syringe. To be eligible for enrollment the subject must have recorded a response of 20 mm on a 100-mm VAS scale on at least one tooth with exposed buccal cervical dentin.

Exclusion criteria were a history of adverse effects with the use of any oral hygiene products; use of over-the-counter desensitizing products within the previous 3 months; use of medications that could interfere with the perception of pain including chronic use of anti-inflammatory, analgesic, anticonvulsant, sedative or other psychotropic drugs; and pregnancy or breastfeeding. Subjects were also excluded from participation if the sensitive tooth was associated with periodontal abscess, mobility >1, or pain from periodontal-related causes or occlusal trauma, or if the sensitive tooth was restored in the previous 3 months or served as an abutment for a fixed or removable dental prosthesis.

### 2.2 Washout Period and Tray Fabrication

Subjects who satisfied enrollment criteria were given a commercially available standard fluoride dentifrice (Colgate Cavity Protection, Colgate-Palmolive) and an adult soft-bristled toothbrush to use for a period of 1 week. Subjects were instructed to use the dentifrice and toothbrush for 2 minutes twice a day in place of their normally used dentifrice and toothbrush. Subjects were given no further instructions regarding oral hygiene during the washout period.

At the time of enrollment (beginning of washout period), impressions of the arch(es) containing the identified sensitive tooth or teeth were made using alginate impression material (Jeltrate Plus, Dentsply Caulk) and disposable plastic trays. Impressions were poured with yellow dental stone (Microstone, WhipMix), and spacer was applied to the dental casts on the buccal surface of identified sensitive teeth to create room for the test or control cream. Custom trays were fabricated using a vacuum former (UltraVac, Ultradent) and 0.9-mm soft tray material (Sof-Tray, Ultradent). Subjects were asked to return after 1 week.

### 2.3 Study Treatment

At the end of the washout period, patients returned to the clinic for baseline sensitivity measurements by a clinical examiner (KL or SS). Ice-cold stimulus was applied using refrigerant spray (Endo-Ice, Hygenic) on a cotton pellet for 1 second. Air stimulus was applied using a dental air-water syringe for 2 seconds. Patient response to ice-cold and air stimuli were recorded on the VAS and DPS pain scales for each sensitive tooth identified at the time of enrollment. To ensure accurate interpretation and analysis of the sensitivity response, the primary (KL) and secondary (SS) examiners were trained and calibrated by a benchmark examiner (the primary investigator BA) on DHS assessment techniques. The first five subjects that were recruited into the study were used for the calibration exercise. These were patients who had been diagnosed as having DHS. The sensitivity evaluation data recorded during calibration comprised four measurements: Air VAS, Air DPS, Cold VAS, and Cold DPS. The agreement between the two examiners’ objective evaluation of sensitivity (interexaminer agreement) and each examiner’s individual sensitivity evaluation (intraexaminer agreement) was evaluated using the unweighted kappa (κ) statistic.

Patients were then randomized by the study coordinator to a group to use either dental cream containing 20% nanohydroxyapatite (‘nHAP’) or dental cream containing 20% pure silica, in the form of standard pure silica particles combined with hydrated nanosilica (‘Silica’). However, to ensure that both the operators and the patients were blinded as to product assignment, all dental cream tubes were packaged identically and coded (P or Q) by the manufacturing/ packaging company, who retained the code until the completion of the study and data interpretation. The custom trays were tried in place and assessed for fit and comfort.

Patients were instructed to continue brushing with the standard fluoride toothpaste and toothbrush for 2 minutes in the morning and 2 minutes before bed, followed on each occasion by rinsing with 10 mL of water for 10 seconds. Patients were instructed to apply their respective cream for 5 minutes once daily following the before-bed brushing using a ribbon of cream in the custom tray, followed by expectoration of the cream without rinsing. The first cream-use occasion took place at the clinical research facility and was supervised by the study coordinator. Subjects were instructed not to take any food or drink for 30 minutes after cream use. As a method of monitoring compliance, a calendar was provided to each subject for recording of daily cream use, and in addition, dental cream tubes were weighed at the time of randomization and at each study visit. Subjects were instructed to maintain their normal dietary habits and were prohibited from using any other oral hygiene product (*e.g*., mouthwash, chewing gum) or tooth-whitening product for the duration of the study.

Subjects returned to the clinical research facility after 2, 4, 6, and 8 weeks. At each visit, response to ice-cold and air stimulus was recorded in four measurements (Air VAS, Air DPS, Cold VAS, and Cold DPS) as described above, and a clinical examiner screened the subjects for adverse events.

### 2.4 Power Analysis and Sample Size Calculation

The sample size calculations, which were based on a power analysis, were performed using nQuery Advisor software (Statistical Solutions). Based on previous studies, [46, 47] and for the null hypothesis that each of the two creams promotes relief of DHS that is significantly greater than zero, an effective sample size of 25 subjects in each treatment group would have power greater than 0.95 with a 0.05 one-sided significance level to detect a difference between a null hypothesis mean of zero and a sample mean percent change in VAS or DPS equal to or greater than 10% using a two-sample *t*-test. The primary analysis was a linear mixed effect model, which should achieve similar or greater power as the two-sample t-test. The primary outcome to be examined in the present study was disease treatment, *i.e*., relief of DHS.

### 2.5 Statistical Methods

The VAS and DPS values as well as their respective percent changes compared to baseline were treated as continuous variables and assessed separately using a random-intercept mixed model analysis that accounted for the correlation among outcomes from the same patient. The primary test of the treatment effect was the treatment by time interaction. Additionally, all the mean treatment outcomes at each time point were compared with the Tukey Honestly Significant Difference (HSD) test for multiplicity. The secondary test of the treatment effect was a *t* test between treatment groups using final (8 weeks) data. A *P* value of < .05 was considered significant. Statistical analyses were conducted using the R environment for statistical computing (v3.1).

## 3 RESULTS

A flow diagram of participation in the trial is shown in Fig. (**1**). Of the 56 subjects recruited for this trial, 4 declined to participate further during the washout period. One subject declined to participate further after randomization but prior to the first posttreatment sensitivity measurement. Fifty-one healthy adults (41 females, 10 males) with a mean (SD) age of 45.5 (13.0) years, from different ethnic origins and socioeconomic statuses, participated in this study (Table **1**). The number of participants enrolled in each group were as follows: nHAP (n = 25) and Silica (n = 26). The mean sensitivity at baseline for each measurement (Air VAS, Air DPS, Cold VAS, and Cold DPS) is shown in Table **1** for the nHAP and Silica groups. No incidences of adverse effects were reported by subjects or ascertained clinically.

The intraexaminer agreement values (unweighted κ value) for the four sensitivity measurements for the primary (KL) and secondary (SS) examiners respectively were 0.80/0.88 (Air VAS), 0.87/0.89 (Air DPS), 0.91/0.94 (Cold VAS), and 0.90/0.89 (Cold DPS). The mean interexaminer reliability values for the four sensitivity measurements comparing the benchmark examiner versus each of the two examiners (KL and SS) were 0.72 (primary) and 0.80 (secondary). These values met the targets set for acceptability prior to the training and calibration.

**Fig. (2):**
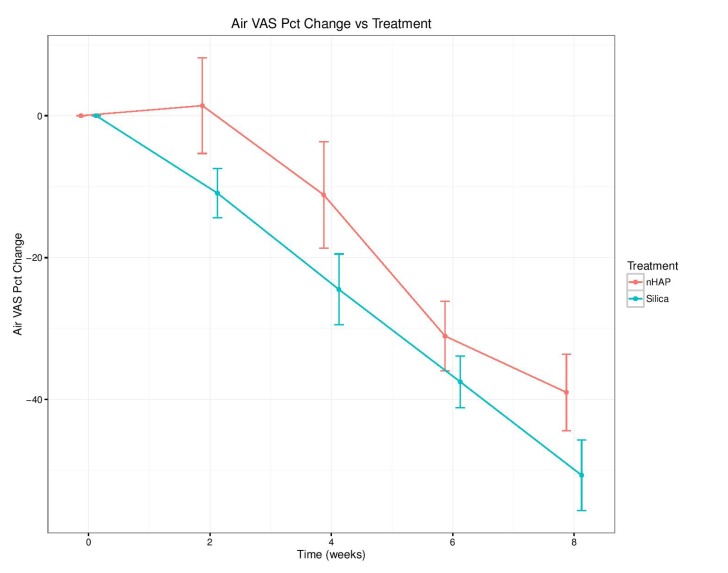
Percent change from baseline over time for Air VAS. There is a significant (*P* < .001) reduction in DHS at each time point with either nHAP or Silica, but there is no significant difference in DHS reduction between nHAP and Silica.

**Fig. (3):**
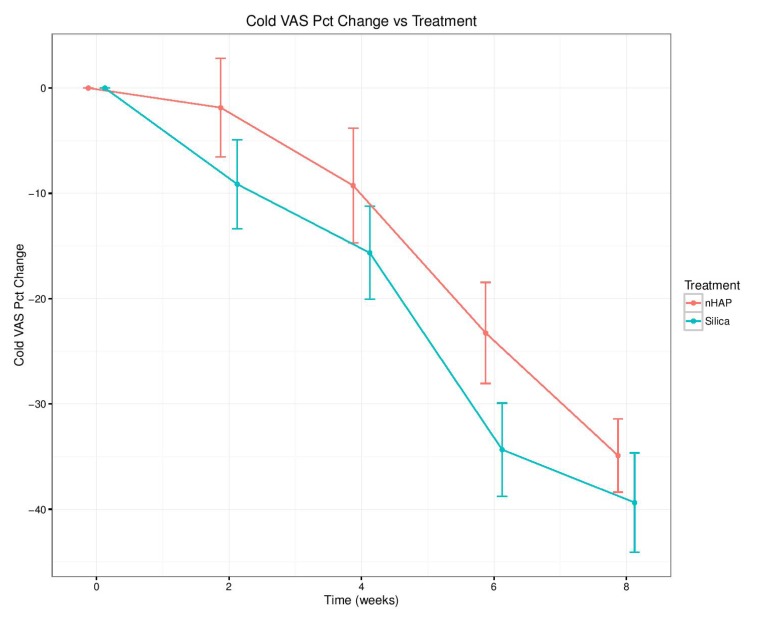
Percent change from baseline over time for Cold VAS. There is a significant (*P* < .001) reduction in DHS at each time point with either nHAP or Silica, but there is no significant difference in DHS reduction between nHAP and Silica.

**Fig. (4):**
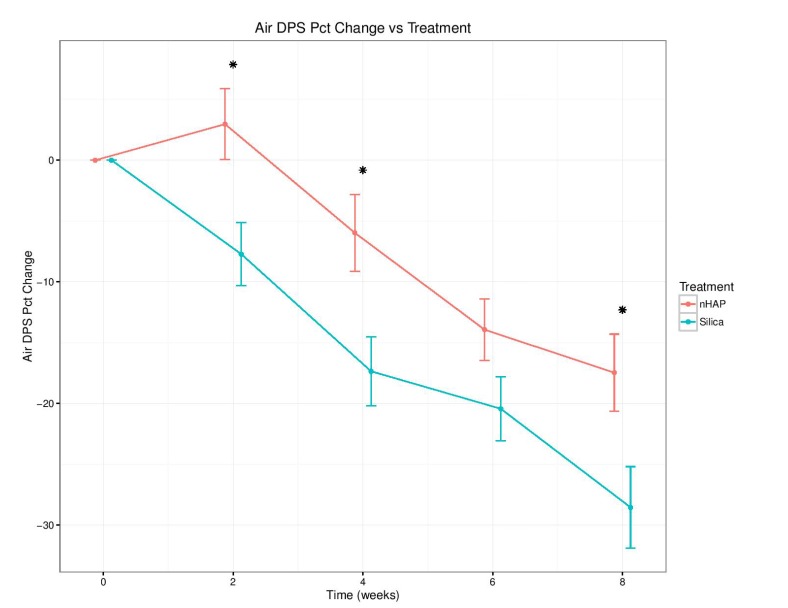
Percent change from baseline over time for Air DPS. There is a significant (*P*<.001) reduction in DHS at each time point with either nHAP or Silica, and DHS reduction was significantly (*P*<.05) better for the patients using Silica than those using nHAP at 2, 4, and 8 weeks * *P*<.05.

**Fig. (5):**
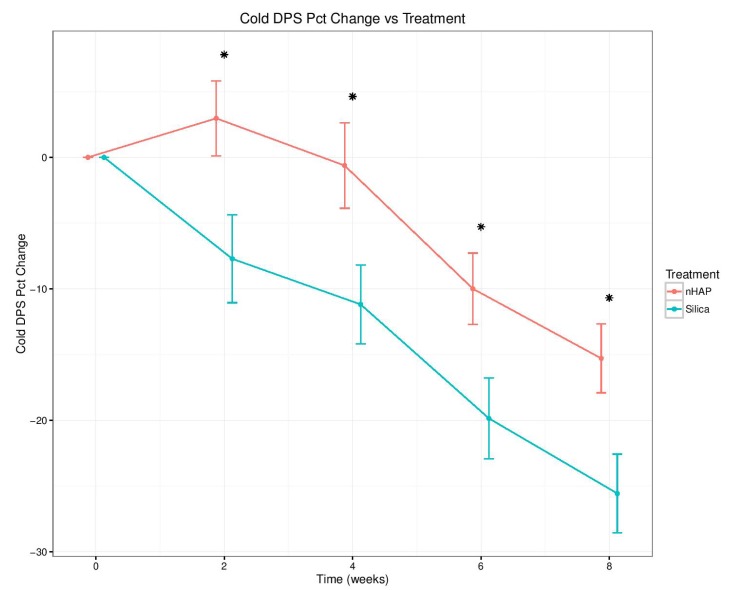
Percent change from baseline over time for Cold DPS. There is a significant (*P*<.001) reduction in DHS at each time point with either nHAP or Silica, and DHS reduction was significantly (*P*<.05) better for the patients using Silica than those using nHAP at all time points * *P*<.05.

The percent change from baseline over time for the four sensitivity measurements is shown in Fig. (**2**-**5**) and in Table **2**. With either air or cold stimulus, VAS and DPS indicated significant (*P* < .001) reduction in DHS at each time point with either nHAP or Silica. VAS showed no significant difference between nHAP and Silica in DHS reduction with either air or cold (Figs. **2** and **3**), although at 8 weeks, Silica had an 11.68% (95% confidence interval [CI], –26.17 to 2.81) larger Air VAS change from baseline and a 4.46% (95% CI, –16.02 to 7.11) larger Cold VAS change from baseline than nHAP. However, when measured with DPS, DHS reduction was significantly (*P* < 0.05) better for the subjects using Silica cream than for those using nHAP cream at all time points with cold (Fig. **5**), and at 2, 4, and 8 weeks with air (Fig. **4**). At 8 weeks, Silica had an 11.07% (95% CI, –20.18 to –1.96) larger Air DPS change from baseline and a 10.27% (95% CI, –18.13 to –2.42) larger Cold DPS change from baseline than nHAP.

## 4 DISCUSSION

The ideal method for reduction of the symptoms of dentin hypersensitivity remains unknown. However, recent reports indicate that mechanical occlusion of the dentin tubules to decrease dentin permeability and thus reduce the excitable impact on the nerves by preventing fluid movement is the most prevalent therapeutic approach, offering fairly rapid results [9, 19-26]. Nanohydroxyapatite is a bioactive and biocompatible material with wide applications in both medicine and dentistry [38, 39]. The effectiveness of an Apadent Pro 20% nHAP-containing topical cream to relieve DHS was investigated in this clinical trial, based on percent change of sensitivity from baseline for four sensitivity measurements: response to an ice-cold stimulus on VAS and DPS, and response to air stimulus on VAS and DPS. Ice-cold and air stimuli were used as the pain provocation tests based on the consensus report on the guidelines for the design and conduct of clinical trials on dentin hypersensitivity [48]. Considering that many stimuli will cause dentinal pain, but not all are suited for quantifying DHS [49, 50], the guideline-recommended tactile, cold and evaporative air stimuli as the stimuli of choice, as these are both physiological and controllable [48]. Furthermore, considering that often individuals will not respond to all types of stimulus or may respond differently to different stimuli [49, 51, 52], it is recommended that at least two hydrodynamic stimuli should be used [46]. However, the results of our pilot study (unpublished) demonstrated ice-cold and air stimuli to be the most sensitive among the three stimuli.

In previous related studies [46, 53-55], following baseline measurement, the time intervals for subsequent measurements of pain intensity varies from weekly, biweekly and monthly, with the use of product extending up to 8 weeks. Thus biweekly time intervals were used in the present study, to cut across the intervals used in previous studies, to enable comparison with the previous related studies.

The findings of the present study rejected the null hypotheses that VAS and DPS scores of the patients treated with Apadent Pro or pure Silica were not significantly different from those before treatment. However, it failed to reject the null hypothesis that there was no statistically significant difference between the VAS or DPS scores of patients treated with Apadent Pro and pure silica in relieving DHS. The percent change from baseline for any sensitivity measurement following 8 weeks of product use ranged from 15.30% (Cold DPS) to 38.99% (Air VAS) for nHAP, and from 25.57% (Cold DPS) to 50.67% (Air VAS) for Silica. Using the VAS pain scale, no significant difference in DHS reduction between the two creams with either air or cold was observed. Using the DPS pain scale, although DHS reduction was significantly better with Silica than nHAP at all time points with cold, the difference was significant only at 2, 4, and 8 weeks with air. It should be noted that the percent change from baseline was smaller at every time point for both air and cold when recorded using the DPS scale as compared to VAS (Figs. **2**-**5** and Table **2**). This may be explained by the restrictiveness [48] of the four-point DPS scale in which participants are limited to the descriptors “none, mild, moderate, or severe” in categorizing their pain. There is currently no single objective method to quantify the subjective patient response to DHS stimuli [56]; therefore the present study adopted the approach of using two pain scales, VAS and DPS. Besides, patient's perception of DHS is subjective and clinical evaluation based on any scoring or rating system regarding its severity is challenging. Nevertheless, it is important to detect, rate and monitor the pain as accurately as possible in order to define the baseline status and to observe any subsequent changes following therapy, so the use of two scales becomes necessary to ensure accuracy. The VAS scale has been shown to be both reliable and valid for the measurement of clinical pain [44], and it has been shown to discriminate among various treatments and changes in pain intensity [45]. The DPS scale, on the other hand, may not be precise enough to allow patients to adequately represent their pain, complicating the process of assessment. Moreover, there is not necessarily an equal interval between the four descriptors, which limits the conclusions that can be drawn about the magnitude of differences between patients or over time [45].

The results of the present study were not unexpected considering the reports of previous studies on nHAP and Silica. An in situ study showed that nHAP pastes resulted in the precipitative occlusion of dentin tubules [43]. The study showed that nHAP and a positive control paste containing silica-based bioglass (NovaMin) were equally effective, and more effective than fluoride pastes, in occluding tubules. The ability of nHAP to deposit a precipitate layer over and within dentin tubules accounts for the relief of bleaching-related sensitivity by nHAP paste reported in a clinical study by Browning *et al*. [54], as well as the significant reduction in DHS reported by Vano *et al*. for patients brushing with nHAP paste as compared to fluoride or placebo paste [53]. However, neither of these clinical studies included a positive control group.

In the present study nHAP-containing cream was compared with a positive control cream containing pure silica, in the form of standard pure silica particles combined with hydrated nanosilica. Similar to nHAP, *in vitro* SEM analysis has shown that mesoporous silica nanoparticles almost completely occlude dentin tubules, forming a seal that penetrates about 105 μm into the tubules [26]. Silica has hydroxyl groups that may bind it to calcium affinity site on dentin [57]. In addition to their ability to enter dentin tubules 2 to 3 µm in diameter, nanoparticles have a high surface energy and large surface area that promote their deposition onto the irregular tubule surface [58, 59]. Dentifrices containing nanosilica in combination with other agents have been studied both *in vitro* and clinically [24, 25, 27], and an *in vitro* study of a biocomposite of nHAP and mesoporous silica nanoparticles showed no significant difference between the percent occluded area for the nHAP/silica biocomposite and the silica nanoparticles alone [27]. This is consistent with the findings of the present study in which the VAS scale showed no significant difference in DHS reduction between the nHAP and Silica creams with either air or cold stimuli.

Other studies have shown a similar effect of reduction in hypersensitivity for nHAP-containing dentifrices as compared to positive controls [55, 60]. Results of a comparison of nHAP home-care paste with an 8% arginine home-care paste and professionally applied sodium fluoride varnish showed no significant difference between the treatments at either 1 or 3 months, with all agents providing clinical reduction in DHS compared with baseline at 1 month, and nHAP and arginine pastes providing DHS relief at 3 months [60]. Golpinath and colleagues reported no significant differences between nHAP and NovaMin bioglass-containing pastes in the response to air, cold, and tactile stimuli at 4 weeks [55].

The high number of subjects assessed but excluded from participating in the study (n = 71) should not be considered a limitation of the study. The most common reason for failure to qualify for study enrollment was lack of exposed buccal cervical dentin among potential participants who self-identified as having tooth sensitivity.

The use of the DPS pain scale may represent a limitation of the present study. The DPS is a four-point verbal descriptor scale, and it has been noted that the averaging of responses on categorical scales may not be meaningful [46]. The percent change from baseline was smaller at every time point for both air and cold when recorded using the DPS scale, as compared to VAS, suggesting that use of the two pain scales, one categorical (DPS) and the other continuous (VAS), to record patient responses to air and cold stimuli may have affected the reliability of the measurements.

Moreover, the effectiveness of the 20% nHAP cream relative to other currently marketed desensitizing agents was not assessed in this study, which showed that Apadent Pro nHAP dental cream and a pure silica positive control cream were equally effective at promoting relief of DHS symptoms. However, nHAP has been shown to have additional properties that may benefit oral health. Nanoparticulate hydroxyapatite may act as a calcium and phosphate reservoir [39], maintaining a topical state of supersaturation of these ions with respect to tooth minerals and causing their deposition on the surface of demineralized tooth tissue. In an *in situ* study nHAP caused the remineralization of early caries lesions [37].

Furthermore, nHAP-containing paste has been shown clinically to decrease the duration of postbleaching sensitivity [54], a common side effect of the bleaching process [61]. Because patients bleaching their teeth at home have been provided with a tray, application of the nHAP cream is a convenient treatment for patients experiencing sensitivity. Future studies on nHAP as a desensitizing agent may focus on the ideal concentration, formulation, or time of application to promote the greatest relief of symptoms for patients with sensitivity secondary to exposed buccal cervical dentin as well as those who develop postbleaching sensitivity.

## CONCLUSION

Both VAS and DPS pain scales indicated significant (*P*<.001) reduction in DHS to air or cold stimuli with either nHAP or Silica. The VAS scale showed that Apadent Pro dental cream containing 20% nHAP and a 20% pure silica positive control cream were equally effective at promoting relief of DHS symptoms. In contrast, the DPS scale showed statistically superior results for the pure silica positive control cream over 20% nHAP. Within the limits of this study, it can be concluded that 20% nHAP dental cream is an effective method to promote the relief of DHS symptoms when applied daily.

## Figures and Tables

**Fig. (1):**
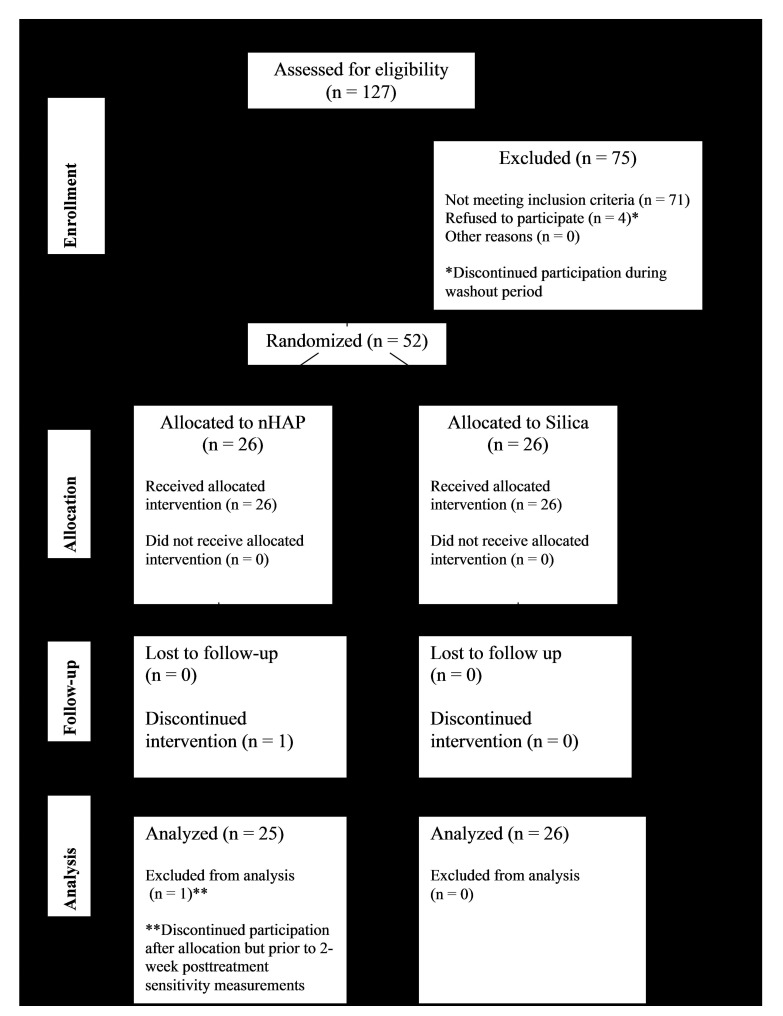
Patient flow diagram.

**Table 1:** Subject demographics and mean sensitivity at baseline.

–	**nHAP**	**Silica**	**Total**	***P* Value**
**Sex**	–	–	–	0.5
Female	19 (76%)	22 (84.62%)	41 (80.39%)	–
Male	6 (24%)	4 (15.38%)	10 (19.61%)	–
Total	25	26	51	–
**Age**	–	–	–	0.02
Mean ± SD	41.4 ± 14.14	49.38 ± 10.62	45.47 ± 13	–
**Ethnicity**	–	–	–	0.65
Asian or Pacific Islander	1 (4%)	3 (11.54%)	4 (7.84%)	–
Black (not Hispanic)	4 (16%)	4 (15.38%)	8 (15.69%)	–
Hispanic	13 (52%)	14 (53.85%)	27 (52.94%)	–
Other	0 (0%)	1 (3.85%)	1 (1.96%)	–
White (not Hispanic)	7 (28%)	4 (15.38%)	11 (21.57%)	–
Total	25	26	51	–
Average sensitivity per subject at baseline* (Mean ± SD)	–	–	–	–
Air VAS	50.32 ± 19.4	51.51 ± 19.12	50.93 ± 19	0.93
Cold VAS	60.47 ± 17.64	65.09 ± 21.53	62.83 ± 20	0.36
Air DPS	2.66 ± 0.63	3.04 ± 0.58	2.85 ± 1	0.05
Cold DPS	3.12 ± 0.5	3.32 ± 0.56	3.22 ± 1	0.11

*VAS score, 0 to 100; DPS score, 0 to 4.

**Table 2:** Percent change in sensitivity from baseline.

**Variable**	**Time (Weeks)**	**Silica (%)**	**nHAP (%)**	**Difference** **Silica–nHAP (%)**	**CI**	***P* Value**
Air VAS	2	–10.93	1.42	–12.35	[–27.66, 2.97]	0.1132
	4	–24.48	–11.15	–13.33	[–30.88, 4.22]	0.1355
	6	–37.51	–31.06	–6.45	[–18.47, 5.57]	0.2906
	8	–50.67	–38.99	–11.68	[–26.17, 2.81]	0.1132
Cold VAS	2	–9.14	–1.86	–7.28	[–19.83, 5.28]	0.2539
	4	–15.65	–9.26	–6.39	[–20.13, 7.36]	0.3597
	6	–34.34	–23.26	–11.08	[–23.98, 1.81]	0.0913
	8	–39.36	–34.90	–4.46	[–16.02, 7.11]	0.4472
Air DPS	2	–7.72	2.97	–10.69	[–18.44, –2.94]	0.0072*
	4	–17.35	–5.97	–11.38	[–19.73, –3.04]	0.0079*
	6	–20.93	–13.93	–6.50	[–13.74, 0.73]	0.0776
	8	–28.54	–17.47	–11.07	[–20.18, –1.96]	0.0176*
Cold DPS	2	–7.72	2.97	–10.69	[–19.33, –2.04]	0.0158*
	4	–11.19	–0.62	–10.57	[–19.30, –1.83]	0.0181*
	6	–19.86	–10.0	–9.86	[–18.00, –1.72]	0.0179*
	8	–25.57	–15.30	–10.27	[–18.13, –2.42]	0.0107*

CI = Confidence interval *P < .05
